# Moderate-to-severe hypercalcemia secondary to primary hyperparathyroidism refractory to conventional treatment (therapeutic management with denosumab): case report and literature review

**DOI:** 10.11604/pamj.2025.50.61.46684

**Published:** 2025-02-26

**Authors:** Manuel Carpio Salmerón, Mariano Tébar Caballero, Luis Marin Martinez, Cristian Marco-Alacid

**Affiliations:** 1 Servicio de Endocrinología y Nutrición, Hospital General Universitario Santa Lucía, Cartagena, España; 2 Servicio de Medicina Interna, Hospital General Universitario Reina Sofía, Murcia, España; 3 Sección de Endocrinología y Nutrición, Hospital Virgen de los Lirios, Alcoy, Alicante, España

**Keywords:** Denosumab, hypercalcemia, primary hyperparathyroidism, parathyroid hormone, case report

## Abstract

Severe hypercalcemia resistant to conventional medical treatment is a rare complication of primary hyperparathyroidism, and published information on the use of denosumab in patients with this condition is limited. This article aims to review the current literature on the utility of denosumab as a hypocalcemic agent in the context of a real clinical case. We present the case of an 87-year-old woman with nonspecific symptoms of constipation and asthenia who was diagnosed with severe hypercalcemia secondary to primary hyperparathyroidism. Treatment with cinacalcet, bisphosphonates, and hydration was ineffective, so denosumab was used until surgery. Denosumab stabilized calcium levels, improved renal function, and alleviated the patient's symptoms. Our study highlights that denosumab is a useful tool in managing hypercalcemia associated with primary hyperparathyroidism, either as a preoperative measure or when surgery is contraindicated.

## Introduction

Primary hyperparathyroidism (PHPT) results from excessive secretion of parathyroid hormone (PTH) [1]. It is the leading cause of hypercalcemia in outpatient settings, while cancer is the most common etiology in hospitalized patients. Together, these two etiologies account for approximately 90% of hypercalcemia cases [2]. Approximately 80% of patients with primary hyperparathyroidism present with a single adenoma, whereas 15-20% exhibit multiglandular involvement in the form of hyperplasia or, less frequently, multiple adenomas [3]. Parathyroid carcinoma accounts for less than 1% of cases. Hypercalcemia is classified as mild when serum calcium levels are <12 mg/dL, moderate when between 12-14 mg/dL, and severe when >14 mg/dL [4]. Mild hypercalcemia is associated with nonspecific neurological symptoms, such as difficulty concentrating or drowsiness. Severe hypercalcemia can lead to higher levels of nervous system depression, ranging from stupor to coma [2]. Currently, the most common presentation of primary hyperparathyroidism is an asymptomatic individual diagnosed based on biochemical findings that define the disease [1]. The diagnosis of primary hyperparathyroidism is biochemical. It requires demonstrating hypercalcemia in two separate measurements and elevated or inappropriately normal PTH levels (near the upper limit of normal). It is a diagnosis of exclusion.

The treatment of choice for primary hyperparathyroidism is surgical. Treatment indications are any patient with symptoms or patient without symptoms but have serum calcium >1 mg/dl (0.25 mmol/L) above the upper limit of normal or bone involvement (vertebral fracture detected by bone densitometry or X-ray, or decrease in bone mineral density with a T-score ≤-2.5) or estimated glomerular filtration rate (eGFR) < 60 mL/min or nephrocalcinosis or nephrolithiasis or hypercalciuria (> 250 mg/day in women or > 300 mg/day in men) or age <50 years [5]. Patient preferences in the absence of contraindications [6]. However, not all patients meeting surgical criteria undergo surgery, as medical contraindications, persistent hyperparathyroidism after surgery by an experienced surgeon, or patient refusal may preclude the procedure [6]. In any case, surgery remains an individualized decision shared with the patient. In situations where surgery is not feasible or is declined by the patient, medical treatment is the preferred option. Cinacalcet, a calcimimetic agent, acts by binding to the calcium-sensing receptor (CaSR), reducing PTH release. Cinacalcet has been shown to effectively reduce serum calcium in primary hyperparathyroidism; however, no improvement in bone mineral density has been observed [6].

Intravenous bisphosphonates, such as pamidronate and zoledronate, are first-line agents for the treatment of tumor-related hypercalcemia [4]. In primary hyperparathyroidism, oral alendronate has been proposed. However, unlike cinacalcet, studies to date suggest that alendronate improves bone mineral density and reduces bone turnover rates without consistently lowering serum calcium [6]. Denosumab, another antiresorptive agent, is a human IgG2 monoclonal antibody targeting Rank ligand. Rank ligand is a molecule released by osteoblasts that interacts with a specific receptor (RANK) on osteoclast precursors. The interaction between RANK ligand and RANK promotes the differentiation of precursors into mature osteoclasts. Denosumab is approved for the treatment of tumor-induced hypercalcemia, sharing similar indications with intravenous bisphosphonates. Furthermore, the 2023 guidelines from the Endocrine Society place denosumab as a preferred agent, based on indirect evidence suggesting greater potency than bisphosphonates [4]. The role of denosumab as a therapeutic agent in primary hyperparathyroidism remains to be determined. Current evidence is limited, but data from a retrospective study conducted in 2020 [6] and several case reports [7-9] suggest that some patients may benefit from this treatment. This study aims to shed light on the therapeutic potential of denosumab in primary hyperparathyroidism by describing a real patient case.

## Patient and observation

**Patient information:** an 87-year-old woman was referred on a priority basis to the Endocrinology and Nutrition outpatient clinic due to an incidental finding of moderate hypercalcemia (serum total calcium: 12.6 mg/dL, reference range: 8.8-10.2 mg/dL). The patient´s medical history included grade I hypertension for 15 years, non-insulin-dependent type 2 diabetes, mixed dyslipidemia treated with statins, osteoporosis (T-score < -2.5), and a history of renal colic. Chronic medications included metformin 1000 mg every 12 hours, atorvastatin 40 mg/day, and olmesartan 10 mg/day. The patient was independent in activities of daily living but reported fatigue, asthenia, and constipation for the previous 6-7 months, prompting a primary care consultation.

**Clinical findings:** on physical examination, the patient was conscious, oriented, and in good general condition, appearing normohydrated and normocolored. Vital signs were within normal limits. The body mass index (BMI) was 24 kg/m^2^. A systemic physical examination revealed no abnormalities.

**Timeline of current episode:** overall, the patient presented with constipation, gastrointestinal discomfort, and apparent weakness in both lower limbs. Initial laboratory tests revealed a total calcium level of 12.6 mg/dL and elevated creatinine (1.4 mg/dL, reference range: 0.50-0.99 mg/dL) compared to a previous level of 0.9 mg/dL. The estimated glomerular filtration rate (eGFR) was 40 mL/min (previously 65 mL/min).

**Diagnostic assessment:** the differential diagnosis included primary hyperparathyroidism, prompting a follow-up laboratory assessment. The second blood test confirmed moderate hypercalcemia with a total calcium of 12.2 mg/dL and elevated parathyroid hormone (PTH) levels of 390 pg/mL (reference range: 15-65 pg/mL). Serum phosphate levels were low at 2.1 mg/dL (reference range: 2.5-4.5 mg/dL), while vitamin D levels were normal.

**Diagnosis:** these findings led to the diagnosis of primary hyperparathyroidism.

**Therapeutic interventions:** although the patient met surgical criteria (serum calcium >1 mg/dL above the upper limit, eGFR <60 mL/min, T-score < -2.5), an initial conservative approach was chosen based on the patient´s clinical characteristics and preferences. Treatment with cinacalcet 30 mg/day was started, and adequate hydration was recommended, with outpatient follow-up planned.

**Figure 1:**
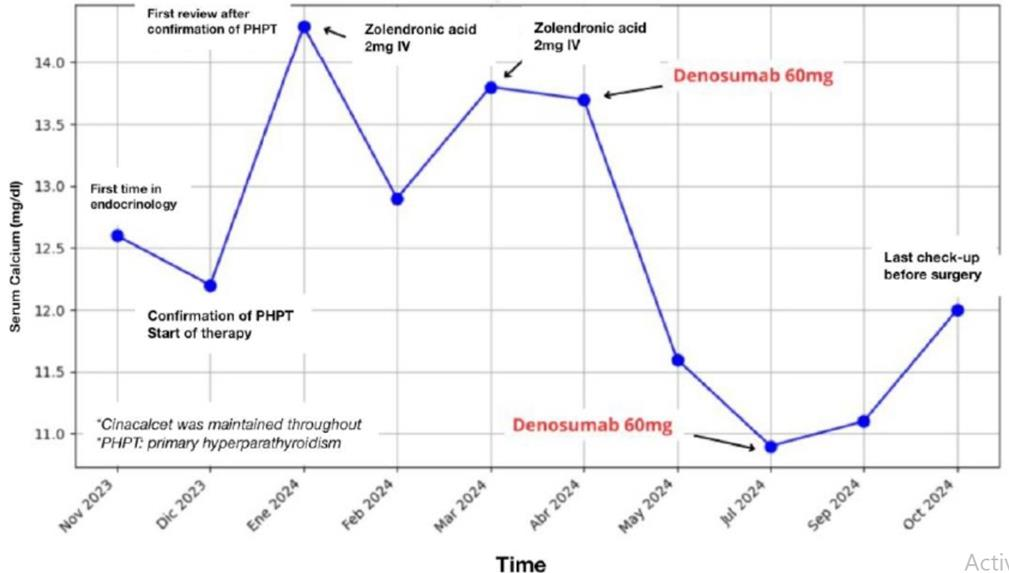
temporal evolution of serum calcium + interventions

**Follow-up and outcome of interventions:** despite cinacalcet therapy, total calcium levels increased to 14.3 mg/dL, and PTH to 450 pg/mL. Intravenous zoledronic acid (2 mg) was added to the regimen alongside continued cinacalcet 30 mg/day. The patient declined hospitalization. Following this, calcium levels decreased to 13 mg/dL, prompting an increase in cinacalcet dosage to 60 mg/day. Despite the increase of cinacalcet (from 30 mg/24h to 60 mg/24h), total calcium was still at 13.8 mg/dl, so a new dose of 2mg iv zoledronic acid was administered and the dose of cinacalcet was reduced to 30 mg/day due to gastrointestinal side effects. Total zoledronic acid administration reached 4 mg, but calcium levels remained elevated at 13.7 mg/dL (Figure 1). Due to inadequate response to medical therapy, imaging studies (thyroid ultrasound and 99mTc-sestamibi scintigraphy) were performed, suggesting a hyperfunctioning parathyroid adenoma. The patient was placed on the surgical waitlist, and subcutaneous denosumab 60 mg was initiated. After administering 60 mg of denosumab, total calcium decreased to 11.6 mg/dL, PTH remained elevated (480 pg/mL), and renal function improved significantly (serum creatinine: 1.1 mg/dL; eGFR: 57 mL/min). Clinically, the patient was asymptomatic, with resolution of gastrointestinal symptoms, asthenia, and restoration of full lower limb strength (5/5). Calcium levels continued to decrease, reaching near-normal levels (serum calcium: 10.9 mg/dL), and renal function normalized (creatinine: 0.9 mg/dL; eGFR: 64 mL/min).

Three months after the initial dose of denosumab, a second 60 mg dose was administered subcutaneously. Two months after the second denosumab dose, total calcium rose to 11 mg/dL, and surgery was scheduled (Figure 1). At the pre-surgical follow-up, the patient had a total calcium level of 12 mg/dL. Complementary imaging studies, including thyroid ultrasound, 99mTc-sestamibi scintigraphy, and single-photon emission computerized tomography (SPECT-CT), were performed. Thyroid ultrasound revealed an enlarged thyroid gland with normal echogenicity, two Thyroid imaging reporting and data system (TIRADS-3) thyroid nodules, and a 7.4x7.2 mm well-defined, hypoechoic, solid mass in the posteromedial region, suggestive of a parathyroid adenoma. Single-photon emission computerized tomography showed posterior prolongation of activity at the lower right pole, corresponding to the nodule described on the prior ultrasound in the posteromedial region of the right lower thyroid. The patient underwent right superior parathyroidectomy and total thyroidectomy. Histopathological findings confirmed a papillary thyroid microcarcinoma in the right lobe and a parathyroid adenoma in the right superior pole. Post-surgery, calcium levels normalized to 9.7 mg/dL, and the patient was completely asymptomatic.

**Patient perspective:** the patient experienced a significant improvement in her symptoms following the administration of denosumab.

**Informed consent:** the patient provided informed consent for the publication of this article. There is no data in the article that could potentially compromise the patient's confidentiality.

## Discussion

In our article, we describe the management of hypercalcemia secondary to primary hyperparathyroidism in an elderly patient. Various pharmacological therapies are detailed, with particular emphasis on the use of denosumab, a drug with limited clinical experience in this condition. Additionally, we present the evolution of analytical parameters in response to the interventions applied. In our patient, cinacalcet was administered as initial therapy at a dose of 30 mg/day, with a maximum dose of 60 mg/day. No significant reduction in total serum calcium was observed; in fact, calcium levels increased (from 12.2 mg/dL to 14.3 mg/dL) three weeks after initiating cinacalcet at 30 mg/day. Furthermore, the 60 mg/day dose was poorly tolerated by the patient. These findings could be explained by the probable early hypocalcemic effect of cinacalcet, which begins within the first 24-48 hours, followed by a decrease in efficacy after the third day. Additionally, the analytical follow-up, conducted every three weeks, may have missed serum calcium fluctuations occurring shortly after treatment initiation. Another significant factor was the inability to appropriately adjust the dose due to the patient´s intolerance to the drug [4]. Another possibility to consider is the formation of antibodies that could limit the activity of cinacalcet. The introduction of zoledronate was associated with an initial decrease in calcium levels; however, this effect was not sustained over time, and the administration of a second dose proved ineffective.

Recent publications have demonstrated that denosumab may play an important role as a hypocalcemic agent. In fact, denosumab has been proposed as a treatment option for PTH-related hypercalcemia, particularly in cases where other therapies have failed. In our patient, the administration of 60 mg of subcutaneous denosumab, in combination with cinacalcet at 30 mg/day, resulted in a significant reduction in total serum calcium, from 13.7 mg/dL to 11.6 mg/dL in three weeks, with near-complete normalization of calcium levels (10.9 mg/dL) three months after administration. These results are consistent with those reported in the literature, such as the denocina study [10], which observed normalization of plasma calcium in patients with hyperparathyroidism treated with a combination of denosumab and cinacalcet. However, one month after the second administration of 60 mg of denosumab, serum calcium levels slightly increased, reaching 12 mg/dL two months after the second dose and prior to surgery. This transient hypocalcemic effect of denosumab has already been described in studies of malignant hypercalcemia, where a loss of long-term efficacy was observed. Similarly, the denocina study reported a significant hypocalcemic response in hyperparathyroidism patients; however, most patients eventually returned to their baseline calcium levels. This may be because denosumab inhibits bone resorption but does not interfere with other pathways by which PTH induces hypercalcemia, such as increased renal hydroxylation of vitamin D, leading to enhanced intestinal calcium absorption and reduced renal calcium excretion. Bone mineral density, on the other hand, remained significantly increased one year after denosumab administration [10].

**Figure 2:**
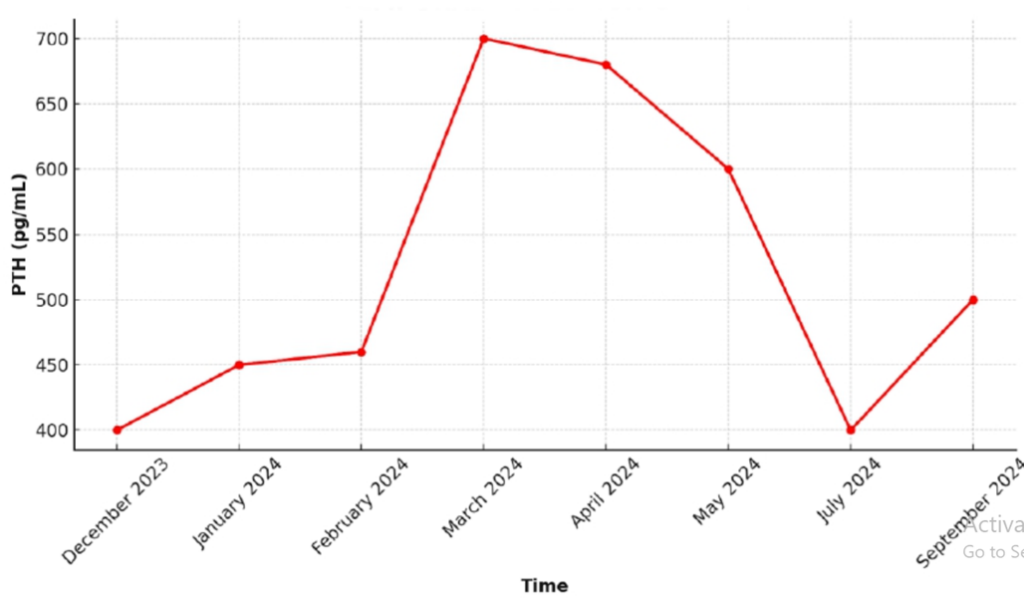
temporal evolution of parathyroid hormone (mg/dl)

Further evidence points to an initial potent hypocalcemic effect of denosumab. A retrospective study published in 2020 reported 10 patients with severe refractory hypercalcemia secondary to hyperparathyroidism treated with denosumab, achieving normalization of calcium levels within 7-9 days in 8 out of 10 patients [7]. Additionally, numerous case reports, particularly in primary hyperparathyroidism secondary to parathyroid carcinoma, have described effective control of hypercalcemia with denosumab [8-9]. The effect of denosumab on PTH levels remains controversial. Several studies show a slight decrease in PTH levels [7]. In the denocina trial, plasma PTH levels initially rose rapidly after denosumab administration, followed by a slow but significant decline [10]. In our patient, as shown in Figure 2, PTH levels remained elevated throughout the clinical course; however, significant changes were observed after denosumab administration. Parathyroid hormone levels decreased by half three months after the initiation of 60 mg of denosumab (from 690 mg/dL to 379 mg/dL), coinciding with the lowest calcium levels (10.9 mg/dL), but later increased despite the second dose of denosumab. Adverse effects of denosumab include hypocalcemia in patients with severe renal disease and jaw osteonecrosis. The latter is also caused by bisphosphonates; however, this side effect is extremely rare and is generally associated with profound and prolonged inhibition of bone resorption, which is not observed with a single injection. Additionally, denosumab has no nephrotoxic effects and does not require dose adjustment in patients with renal insufficiency, unlike bisphosphonates, which must be adjusted according to renal function.

The improvement in renal function observed in our patient can be explained by both active rehydration and a significant reduction in calcium levels. Severe hypercalcemia can cause acute kidney injury through direct renal vasoconstriction and reductions in extracellular fluid volume. The described case and available evidence suggest that denosumab could become a useful therapeutic agent for the medical management of primary hyperparathyroidism. The evidence indicates that denosumab initially achieves a very significant reduction in calcium levels in patients with primary hyperparathyroidism, although there is a subsequent trend toward recurrence of hypercalcemia, while the benefits on bone mineral density may persist over time. Nevertheless, given the limited evidence, it is essential to conduct new, long-term controlled clinical trials with a larger number of participants to better define denosumab´s therapeutic potential in these patients and to improve our understanding of the underlying pathophysiological mechanisms driving its effects.

## Conclusion

The treatment of primary hyperparathyroidism in elderly patients should be individualized, considering both conservative and surgical management as equally valid options, depending on the patient's clinical profile. Denosumab represents an alternative in the medical management of moderate-to-severe hypercalcemia secondary to primary hyperparathyroidism, particularly in patients with renal insufficiency and/or when bisphosphonates are contraindicated. The efficacy of denosumab in reducing hypercalcemia is effective, temporary, and limited, with a tendency for serum calcium levels to recur to initial values, thus not being considered a definitive therapy. Due to its acute and transient effect, denosumab could be used as a “bridge therapy” to achieve temporary normalization of serum calcium levels until definitive surgical treatment for primary hyperparathyroidism is performed.
